# Conciliatory Anti-Allergic Decoction Attenuates Pyroptosis in RSV-Infected Asthmatic Mice and Lipopolysaccharide (LPS)-Induced 16HBE Cells by Inhibiting TLR3/NLRP3/NF-*κ*B/IRF3 Signaling Pathway

**DOI:** 10.1155/2022/1800401

**Published:** 2022-09-29

**Authors:** Ya-qin Chen, Yan Zhou, Qi-li Wang, Jian Chen, Hua Chen, Hui-hui Xie, Lan Li

**Affiliations:** ^1^Department of Paediatrics, The First Affiliated Hospital of Zhejiang Chinese Medical University, Zhejiang, China; ^2^The First Clinical Medical College, Zhejiang Chinese Medical University, Zhejiang, China; ^3^Department of Hospital Management, Zhejiang Chinese Medical University, Zhejiang, China

## Abstract

Respiratory syncytial virus (RSV) infection can deteriorate asthma by inducing persistent airway inflammation. Increasing evidence elucidated that pyroptosis plays a pivotal role in asthma. Conciliatory anti-allergic decoction (CAD) exhibits an anti-inflammatory effect in ovalbumin (OVA)-induced asthma; however, the effects and mechanisms of CAD in RSV-infected asthmatic mice have not yet been elucidated. The RSV-infected asthmatic mice model and lipopolysaccharide (LPS)-induced 16HBE cell pyroptosis model were established, respectively. Pulmonary function, ELISA, and histopathologic analysis were performed to assess the airway inflammation and remodeling in mice with CAD treatment. Furthermore, ultra-performance liquid chromatography-quadrupole time-of-flight/mass spectrometry (UPLC-Q-TOF/MS) was conducted to identify the chemical compounds of high-dose CAD (30 g/kg). Cell viability and apoptosis of 16HBE cells were assessed by CCK-8 and flow cytometry assays, respectively. Finally, the expression levels of apoptosis-, pyroptosis-, and TLR3/NLRP3/NF-*κ*B/IRF3 signaling-related genes were measured with qRT-PCR or western blotting, respectively. Pulmonary function tests showed that CAD significantly ameliorated respiratory dysfunction, airway hyperresponsiveness, inflammation cell recruitment in BALF, pulmonary inflammation, collagen deposition, and cell death in lung tissues. CAD significantly decreased the content of TNF-*α*, IL-13, IL-4, IL-1*β* and IL-5 in the bronchoalveolar lavage fluid (BALF), IL-17, IL-6, and OVA-specific IgE in serum and increased serum IFN-*γ* in asthma mice. The results of UPLC-Q-TOF/MS showed that high-dose CAD had 88 kinds of chemical components. In vitro, CAD-contained serum significantly suppressed LPS-induced 16HBE cell apoptosis. Additionally, CAD and CAD-contained serum attenuated the up-regulated expressions of Bax, Cleaved caspase-3, NLRP3, ASC, Cleaved caspase-1, GSDMD-N, IL-18, IL-1*β*, TLR3, p-P65, p-I*κ*B*α*, and IRF3 but increased Bcl-1 and GSDMD levels in the asthma mice and LPS-induced 16HBE cells, respectively. These results illustrated that CAD may have a potential role in improving airway inflammation and pyroptosis through inhibition of the TLR3/NLRP3/NF-*κ*B/IRF3 signaling pathway.

## 1. Introduction

Asthma is a chronic inflammatory respiratory disease, and its prevalence and incidence are observably increased year by year, particularly in children [1, 2]. It is widely recognized that the typical features of asthma are chronic airway inflammation, airway hyperresponsiveness, and different degrees of structural remodeling events in the airway and lungs [3, 4]. This heterogeneous disease is usually caused by genetic or environmental factors, such as allergen exposure and infections. Among them, respiratory syncytial virus (RSV) infection is the primary cause of acute asthma in children, whereby ~85% of acute asthma are involved in RSV infection [5]. Currently, glucocorticoids and *β*-2 adrenergic receptor agonists are now considered a standard asthma treatment strategy, and most of the symptoms are significantly improved, including wheezing, shortness of breath, and coughing [4]. Unfortunately, there is low efficacy in chronic and persistent asthma, and there are multiple side effects to high dose or prolonged use. Additionally, there is no impactful therapy for RSV-induced asthma. Hence, there is an urgent need to identify the physiopathologic events of RSV-induced asthma and develop a promising therapeutic avenue for asthma.

Pyroptosis is an inflammatory form of cell death that is related to the formation of a multiprotein complex called the inflammasome [6]. The inflammasome consisted of caspase 1 which plays a central role in cleaving and activating the precursor forms of IL-1*β* and IL-18, and also caspase 1 cleaves gasdermin D (GSDND) to generate N-terminal fragments that form membrane pores that result in cell death [7, 8]. Of interest, the NLRP3 inflammasome has been reported to initiate pyroptosis, and the persistent NLRP3 activation is involved in severe asthma pathogenesis [9]. Recently, a study by Chen et al. reported that NLRP3 inflammasome and pyroptosis inhibition were shown to improve asthma in rats [10]. In addition, pyroptosis of bronchial epithelial contributes to hyperresponsiveness and airway inflammation in toluene diisocyanate-induced asthma through activation of NLRP3 inflammasome and GSDND cleavage [11]. More importantly, RSV induces lytic cell death in macrophages specifically through NLRP3 inflammasome activation [12].

Besides, The innate immune system contributes to the control of RSV infection [13, 14], since it is equipped with a good range of pattern-recognition receptors (PRRs), including Nod-like receptors (NLRs) and Toll-like-receptors (TLRs), that can rapidly induce cytokine release and inflammation control [15, 16]. TLRs are found that contributed to the production of inflammatory mediators via NF-*κ*B by identifying a variety of pathogen-associated molecular patterns (PAMPs) and by acting as a primary signal to evoke NLR's activation [17]. Among the TLRs family, TLR3, a pattern-recognition receptor, recruits TRIF and triggers a strong antiviral response via IRFs [18]. On the other hand, TLR3 contributes to the activation of NF-*κ*B and interferon regulatory factor 3 (IRF3) to induce a series of inflammatory cytokine production in RSV-infected epithelial cells [19, 20] or RAW264.7 macrophages [21]. For this reason, NLRP3 which regulates pyroptosis-induced asthma requires to be further studied, and TLR3/NLRP3/NF-*κ*B/IRF3 signaling inhibition could be an anti-inflammatory strategy in airway epithelial injury.

Traditional Chinese medicine has drawn broad attention from scholars, who have focused on multi-factorial disorders, including the pathogenesis of asthma. At present, several herbal medicines were confirmed to ameliorate airway functions in asthmatic mice with RSV infection [5, 22, 23]. According to the clinical experience of the most prestigious veteran practioners of TCM, Conciliatory anti-allergic decoction (CAD) is used extensively for childhood asthma at the Zhejiang Provincial Hospital of Chinese Medicine. Significantly, CAD belongs to an experimental prescription of Chinese medicine and develops according to the Xiaochaihu decoction, which is composed of *Bupleurum chinense* (Chai Hu), *Scutellaria baicalensis Georgi* (Huang qin), *Prince Ginseng* (Tai zi shen), *Rhizoma Pinelliae* (Fa ban xia), *Ephedra sinica* (Ma huang), *Fritillariae Thunbergii Bulbus* (Zhe bei mu), *Salvia miltiorrhiza Bunge* (Dan Shen), *Farfarae Flos* (Kuang dong hua), *CicadaePeriostracum* (Chan yi), and *Glycyrrhizae* (Zhi gan cao). Our previous research found that CAD can inhibit inflammatory cell invasion, goblet cell hyperplasia, and mucus secretion, and observably attenuate the levels of TNF-*α*, IL-4, IL-5, and IL-13 in bronchoalveolar lavage fluid (BALF) of ovalbumin (OVA)-induced asthmatic mice [24]. These data further confirmed that CAD may restrain the development of allergic lung inflammation in asthma. However, the therapeutic effect and specific mechanism of CAD through the TLR3/NLRP3/NF-*κ*B/IRF3 signaling pathway on RSV-infected asthmatic mice are still unclear.

Therefore, we are devoted to exploring the effects of CAD on pyroptosis and the TLR3/NLRP3/NF-*κ*B/IRF3 signaling pathway in OVA/RSV-induced asthma mouse models, which may contribute to promoting understanding of CAD in the treatment of acute asthma with RSV infection.

## 2. Materials and Methods

### 2.1. Preparation of CAD Aqueous Extract

CAD is a compound composed of ten herbs including 6 g *Bupleurum chinense*, 4.5 g *Scutellaria baicalensis Georgi*, 6 g *Prince Ginseng*, 4.5 g *Rhizoma Pinelliae*, 2 g *Ephedra sinica*, 6 g *Fritillariae Thunbergii Bulbus*, 6 g *Salvia miltiorrhiza Bunge*, 6 g *Farfarae Flos*, 6 g *CicadaePeriostracum*, and 3 g *Glycyrrhizae.* All herbs were purchased and fully checked by Pro. Xiao-Feng Yuan (Medical Research Institute of Traditional Chinese Medicine, Zhejiang Chinese Medical University, Zhejiang, China) based on the Chinese Pharmacopoeia 2015, and processed from the Traditional Chinese Medicine Preparation Room of Zhejiang Provincial Hospital of Chinese Medicine. All herbs were mixed and boiled in 500 mL of ddH2O for 1 h to prepare the aqueous extract of CAD. After 1 h, the supernatant was collected, and then another 500 mL of ddH2O was added to the herbs with boiling for an additional 1 h. Next, the acquired supernatant was mixed and concentrated to 1 g/mL with rotary evaporation under reduced pressure at 75°C. Finally, the samples were stored at 4°C until further use.

### 2.2. Animals and Viral Strain

A total of 60 SPF female BABL/c mice, 6-week-old and weighing 20 ± 2 g, were acquired from the Shanghai SLAC Laboratory Animal Co. Ltd., and kept in the animal experimental center of the Zhejiang Chinese Medical University with a standard irradiated chow diet ad libitum and acclimatized for 1 week before experiments. All animal experimental protocols were approved by the Ethics Committee of the Zhejiang Chinese Medical University (Zhejiang, China). The RSV Long was obtained from the Institute of Virology of Zhejiang Provincial Center for Disease Control and Prevention (Zhejiang, China), and propagated in human epithelial carcinoma derived cells (Hep-2). Also, the viral titer was measured using the Reed-Muench method, and the final titers of preparations were in the range of 10^8^ TCID50 (median tissue culture infective dose)/mL in this study.

### 2.3. OVA/RSV-Induced Mice Asthma Model and Treatment

**Figure 1:**
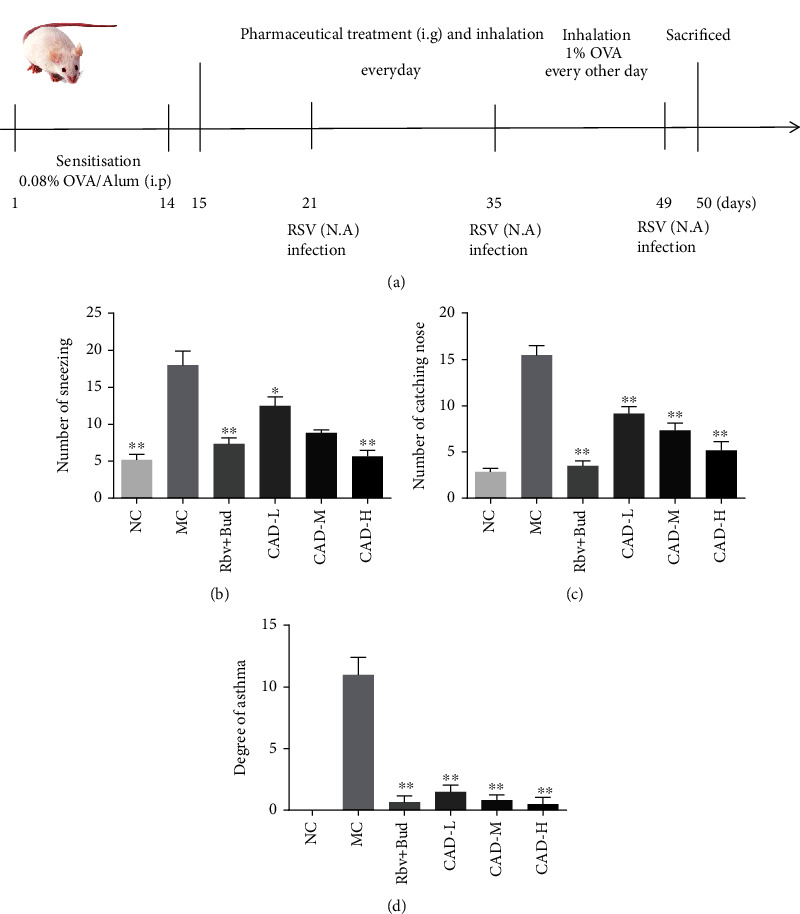
Effect of the conciliatory anti-allergic decoction on the general behavioral characterization of asthmatic mice induced by RSV. (a) Schematic presentation of the OVE/RVS-induced asthma mice model and the pharmaceutical treatment with CAD. The number of sneezing (b), catching nose (c), and asthma (d) of the mice. ^∗^*P < 0.05*, ^∗∗^*P < 0.01*, compared with the MC group.

The BABL/c mice were divided randomly into 6 groups (n =10) as follows: the normal control (NC) group, the asthma model control (MC) group, a ribavirin+budesonide (Rbv + Bud) group, which was used as a positive control; CAD low-dose (CAD-L) group, CAD medium-dose (CAD-M) group, and CAD high-dose (CAD-H) group. The asthma mice model was established as previously published with slight modifications [24]. Except for the NC group, all the mice in the other groups were sensitized on day 1 and day 14 with an intraperitoneal injection containing 100 *μ*g ovalbumin (OVA, Sigma, Saint Louis, MO) together with 2 mg of aluminum hydroxide (Shanghai Xinyu Biotechnology Pharmaceutical Co., Ltd., Shanghai, China) in 200 *μ*l of physiological saline. On days 15-49, the mice were treated every other day with aerosolized 1% OVA in physiological saline for 20 min. On days 21, 35, 49, mice were intranasally administered 50 *μ*L RSV (1 × 10^6^ TCID_50_/mL). From day 15 to day 49, mice in the CAD-L, CAD-M, and CAD-H groups were intragastrically administered CAD at a dose of 7.5 g/kg/d, 15 g/kg/d, and 30 g/kg/d. Mice in the NC and MC groups were administrated with an equal volume of normal saline. The CAD low-dose for mice was determined with a body surface area normalization method according to the normal clinical dose using the following formula: mice (g/kg) = [human dose (50 g crude herbs/day)/human weight (60 kg)] ×9.1. Then, the medium-dose and high-dose CAD were used as 2 and 4 times low-dose of CAD, respectively. Mice in the Rbv + Bud group were intragastrically administered Rbv (FUREN MEDICINES GROUP Co. Ltd. Henan, China) at a dose of 90 mg/kg/d and were challenged with aerosolized Bud (0.2 mg/kg/d, AstraZeneca AB, Sweden) on days 15-49. Except for the Rbv + Bud group, all the mice were treated with aerosolized normal saline for 30 min per day. On the 50^th^ day after the final OVA/RSV challenge, the behavior of the asthma mice as sneezing, catching the nose and the degree of asthma were quantified for a period of 15 min. After that, the pulmonary function studies were performed, then the mice were euthanized to collect blood and lung tissues of all mice for subsequent analysis. A schematic diagram of the OVA/RSV-induction of mice asthma model is illuminated in Figure 1(a).

### 2.4. Measurement of Pulmonary Function

The pulmonary function of mice as directed by breaths per minute (BPM) and tidal volume were assessed using the EMKA animal lung function analysis system (Emka Technologies, France). Additionally, airway hyperresponsiveness was assessed by stimulating with 0, 3.125, 6.25, 12.5, 25, and 50 mg/mL of aerosolized acetylcholine (Shanghai Mengry Biotechnology Co., Ltd., Shanghai, China) by using Whole Body Plethysmography according to the manufacturer's instructions. Then, the enhanced pause (Penh) was used to assess the bronchoconstriction function.

### 2.5. Collection of Bronchoalveolar Lavage Fluid (BALF) and Cell Count

After the completion of the measurement of pulmonary function, the mice were euthanized by cervical dislocation. The lungs were lavaged with cold PBS and then the BALF were initially centrifuged at 2700 g for 10 min at 4°C. Next, the supernatants were collected and frozen at -80°C for further cell count. After that, the cell smear was stained with Wright-Giemsa (Nanjing, China) to assess the number of inflammatory cells, including neutrophils, eosinophils, and lymphocytes under light microscopy (Leica, German).

### 2.6. Enzyme-Linked Immunosorbent Assay

After euthanasia, the abdominal aorta blood samples were taken and then centrifuged at 2700 g for 15 min at 4°C for serum inflammatory factors analysis. Finally, the concentrations of IL-17, IL-6, OVA-specific IgE, and INF-*γ* in the serum, as well as the levels of IL-4, TNF-*α*, IL-13, IL-5, and IL-1*β* in the BALF were measured using commercially available kits (Nanjing, China).

### 2.7. Histopathology Analysis

The lung tissues of each mouse were removed and weighed. Subsequently, the left lung tissues were fixed in 4% paraformaldehyde for 24 h and embedded in paraffin. Then, the tissues were sectioned at 3 *μ*m thickness. After that, the tissue sections were stained with hematoxylin and eosin, Masson's trichrome, PAS, and TUNEL staining to estimate airway inflammation, epithelial injury, collagen deposition, and apoptosis of epithelial lung cells, respectively. Tissue sections were then photographed using light microscopy at 200× magnification. Histological scores were determined as 0 (normal) to 4 (severe): 0: no inflammatory cell; 1: a few inflammatory cells; 2: a thin one-two inflammatory cell layer surrounded in bronchi or vessels; 3: an obvious three to five inflammatory cells layer surrounded in bronchi or vessels, and 4: more than five inflammatory cells layer surrounded in bronchi or vessels [4, 24]. The airway wall thickness and the Masson's trichrome-stained collagen/lumen area were measured with Image-Pro® Plus 6.0 software (Media Cybernetics). Five randomly selected non-overlapping views of per lung tissue section were used to quantify the apoptotic cell levels.

### 2.8. UPLC-Q/TOF-MS Analysis of High-Dose CAD

To identify chemical compositions of high-dose CAD, the UPLC-Q/TOF-MS analysis was performed using an ACQUITY I-Class Plus UPLC system (Waters, USA), including a SCIEX X-500R mass spectrometer (AB SCIEX, USA) and a TurboIonSpray electrospray ionization (ESI) source. Chromatographic separation was performed at 40°C using an ACQUITY UPLC BEH C18 column (100×2.1mm, 1.7*μ*m) with mobile phases A (0.1% formic acid acetonitrile) and B (0.1% formic acid in water). The flow rate was set at 0.3 mL/min. The gradient profile was as follows: 0~6min, 99%B~75% B; 6~13.5min, 75%~65% B; 13.5~15min, 65%~50% B; 15~16.5min, 50%~15% B; 16.5~17min, 15%~1% B; 17~20min, 1% B. Mass spectrometric detection was conducted in electrospray using an IonSapary Voltage Floating of 5.5kV for positive ion mode and -4.5 kV for negative ion mode, the source temperature: 600°C, and the cone gas flow rate, 50L/h. The full scan data were acquired from 100 to 1500 Da. The information-dependent acquisition (IDA) methods were selected to collect secondary mass spectrometry information. Finally, the chemical compositions of high-dose CAD were determined from a self-building TCM MS/MS library of chemical compounds in SCIEX OS software.

### 2.9. Preparation of CAD-Contained Serum

Another 20 SPF male SD rats weighing 180~ 220 g were randomly divided into 4 groups: the control group, CAD low-dose (CAD-L) group, CAD medium-dose (CAD-M) group, and CAD high-dose (CAD-H) group. The CAD groups were orally administered CAD at a dosage of 5, 10, and 20 g/kg twice a day for five days, respectively. The rats in the normal control group were treated with the same amount of normal saline. On the five days, the rats were anesthetized after the last administration for 1 h. After that, the blood samples were collected from the abdominal aorta and centrifuged for 15 min at 3500 rpm and at 4°C to obtain the serum. Next, the serum was inactivated, filtered, and stored at -20°C until subsequent experiments.

### 2.10. Cell Culture

The human bronchial epithelial cell line (16HBE) was purchased from American Type Culture Collection (Manassas, VA). Then, the 16HBE cells were cultured in DMEM (Gibco, Rockville, MD, USA) supplemented with 10% FBS (Gibco), and 1% penicillin/streptomycin (Beyotime, China) in a humidified incubator with 5% CO_2_ at 37°C.

### 2.11. Cell Viability Assay

16HBE cells were counted and seeded into a 96-well plate (3000/well). After 24 h, the cells were treated with LPS (Sigma) at variable concentrations (0, 0.1, 0.5, 1.0, 2.5, 5, 10, 12.5 *μ*g/ml) or serum-free (SF) medium, 10% FBS medium, 10% normal rat serum (NRS) medium, and 10% CAD-contained serum medium for 24 h. Then, 10 *μ*L cell counting kit-8 (CCK-8) solution was directly added to each well and incubation at 37°C for 4 h. The optical density (OD) in each group at 450 nm was detected by using a microplate reader (PerkinElmer, Waltham, MA, USA). To observe the effect of CAD on cell viability in LPS-stimulated 16HBE cells, the cells were divided into 5 groups, respectively, treated with 10% NRS, 10% NRS+ 1 *μ*g/ml LPS, 10% CAD-contained serum medium+1 *μ*g/ml LPS for 24 h. Similarly, cell viability was also determined by the CCK-8 assay.

### 2.12. Flow Cytometry

To investigate the role of CAD on LPS-stimulated cell apoptosis, 16HBE cells were treated with 10% CAD-contained serum medium+1 *μ*g/ml LPS for 24 h. Then, the apoptosis of 16HBE cells was assessed by an Annexin V-FITC/PI apoptosis kit (Beyotime, China) according to the manufacturer's instructions.

### 2.13. Quantitative Real-Time PCR (qRT-PCR)

For the observation of the effect of CAD on lung tissue viral load and TLR3/NF-*κ*B/IRF3 pathway-associated targets, qRT-PCR assay was performed in this study. Total RNA from right lung tissues was isolated using Trizol reagent (Invitrogen, United States) and quantified by NanoDrop (Thermo Fisher Scientific). Immediately afterwards, qRT-PCR was performed using an SYBR®Premix Ex TaqTMII (TAKARA, Japan) on a 7500 FAST Real-Time PCR System (Applied Biosystems, Foster City, CA, USA). Relative mRNA expressions of RSV, TLR3, NF-*κ*B, and IRF3 were normalized to GAPDH levels. The relative expressions were calculated using the 2 ^−*ΔΔ*Ct^ method [25]. The primers used in this study were purchased from Sangon Biotech (Shanghai, China) and shown as followed: RSV, forward: 5´-TCCCATTGATFTCTTFACCA-3´, Reverse: 5´-CCAACGGAGCACAGGAGATA-3´; TLR3, forward: 5´-GTGAGATACAACGTAGCTGACTG-3´, Reverse: 5´-TCCTGCATCCAAGATAGCAAGT-3´; NF-*κ*B, forward: 5´-AGAGCTAATCCGCCAAGCAG-3´, Reverse: 5´-GACCTGTACTTCCAGTGCCC-3´; IRF3, forward: 5´-AGAGGCTCGTGATGGTCAAG-3´, Reverse: 5´-AGGTCCACAGTATTCTCCAGG-3´; GAPDH, forward: 5´-ACAACTTTGGTATCGTGGAAGG-3´, Reverse: 5´-GCCATCACGCCACAGTTTC-3´.

### 2.14. Western Blot Analysis

Total protein of 100 mg of right lung tissues and the treated 16HBE cells were exacted by using cold-RIPA lysis buffer and then the protein was quantified with bicinchoninic acid (BCA) reagent kit (Beyotime). Equal concentrations of protein were separated by 10~15% SDS-PAGE. After transferring to the PVDF membranes (Millipore, USA), the membranes were blocked and overnight incubated with the primary antibodies (Bax, Affinity, 1: 2000; Bcl-2, Affinity, 1 : 1000; Cleaved-Caspase 3, Affinity, 1 : 1000; NRLP3, Abcam, 1 : 1000; ASC, Santa, 1 : 1000; Cleaved-Caspase 1, Affinity, 1 : 1000; GSDMD, Affinity, 1 : 2000; cleaved N-terminal GSDMD, Abcam, 1 : 1000; IL-18, Affinity, 1 : 2000; IL-1*β*, Affinity, 1 : 2000; TLR3, Affinity, 1 : 1000; Phospho-NF-*κ*B p65 (Ser536), Affinity, 1 : 2000; NF-*κ*B p65, Affinity, 1 : 2000; Phospho-IKB alpha (Ser32/Ser36), Affinity, 1 : 2000; IKB alpha, Affinity, 1 : 2000; IRF3, Affinity, 1 : 1000; *β*-actin, Affinity, 1 : 5000) at 4°C. Then, the blots were incubated with corresponding goat anti-rabbit or goat anti-mouse second antibody (1 : 5000, Cell Signaling Technology Inc) at 37°C for 1 h. Ultimately, enhanced chemiluminescence (ECL) agents (Solarbio, China) were used to visualize the protein bands, and the relative protein expression levels were quantified by Image J software (National Institutes of Health).

### 2.15. Statistical Analysis

All data were represented as mean ± SD. The results were by SPSS 22.0 statistical software (SPSS, Inc.). One-way analysis of variance with Tukey's post hoc test was used in more than two groups. *P-value*< 0.05 was considered statistically significant.

## 3. Results

### 3.1. CAD Ameliorated the Pulmonary Function of Asthmatic Mice Induced by RSV

**Figure 2:**
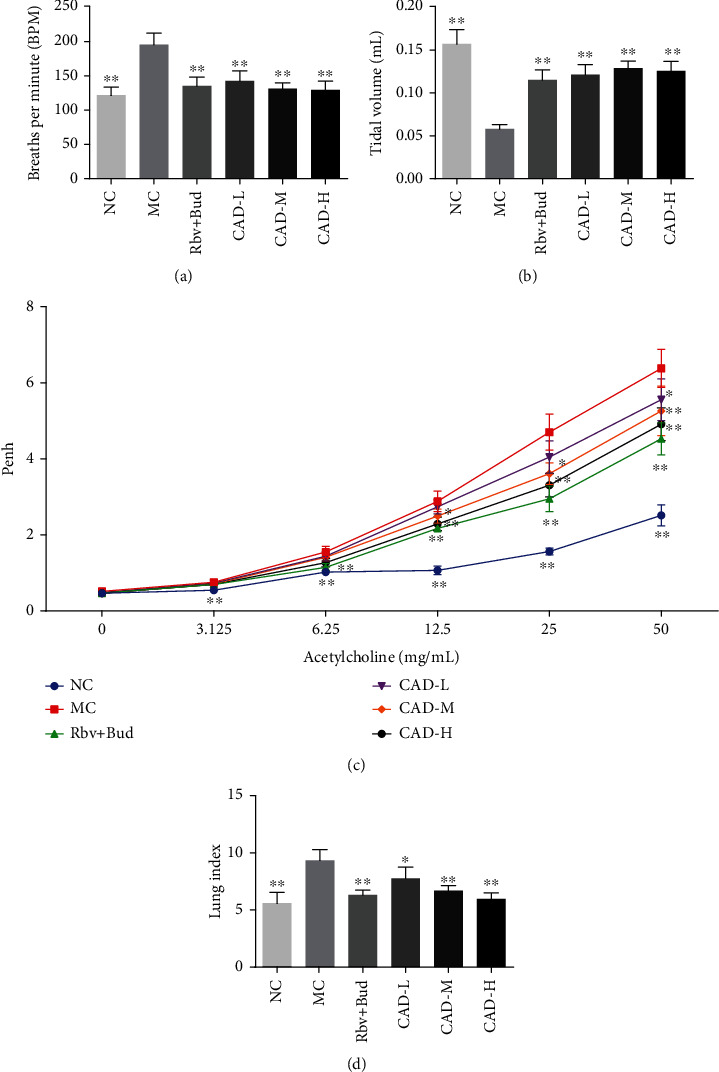
Effect of the conciliatory anti-allergic decoction on the pulmonary ventilation function in asthmatic mice. The breaths per minute (a) and the tidal volume (b) of asthmatic mice were evaluated. (c) Pulmonary responses were determined by whole-body plethysmography. (d) The lung index in asthmatic mice. ^∗^*P < 0.05*, ^∗∗^*P < 0.01*, compared with the MC group.

The mice of the MC group showed an increase in sneezing, catching nose, and degree of asthma as compared to the mice in NC group (Figures 1(b)–1(d)). However, mice treated Rbv + Bud or CAD showed a reduction in the number of sneezing and catching nose in a dose-dependent manner, compared with MC group. Besides, we found that CAD significantly attenuated the BMP levels when compared with MC group (Figure 2(a)), and the tidal volume of mice was markedly increased after CAD treatment (Figure 2(b)). Subsequently, we treated mice with different concentrations of acetylcholine. The results revealed that Penh significantly increased in MC group, and CAD could reverse the airway hyperresponsiveness (Figure 2(c)). These results suggested that CAD effectively improved the pulmonary ventilation function of RSV-infected asthmatic mice.

### 3.2. CAD Decreased Inflammatory Cells and pro-Inflammatory Cytokines in Asthmatic Mice

**Figure 3:**
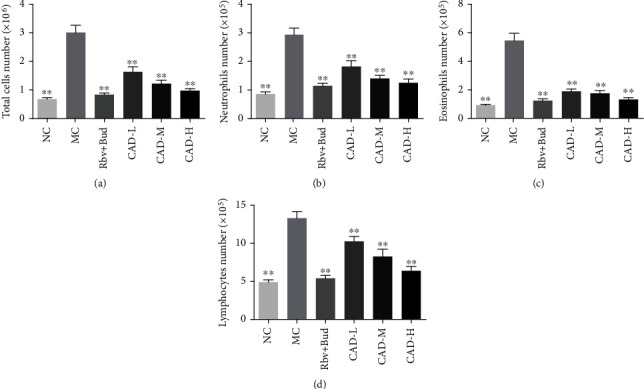
Effect of the conciliatory anti-allergic decoction on inflammatory cells in asthmatic mice induced by RSV. The cell numbers of total cells (a), neutrophils (b), eosinophils (c), and lymphocytes (d) in BALF of mice in the MC group were significantly increased, while that in the CAD-treated groups were significantly decreased. ^∗∗^*P < 0.01*, compared with the MC group.

**Figure 4:**
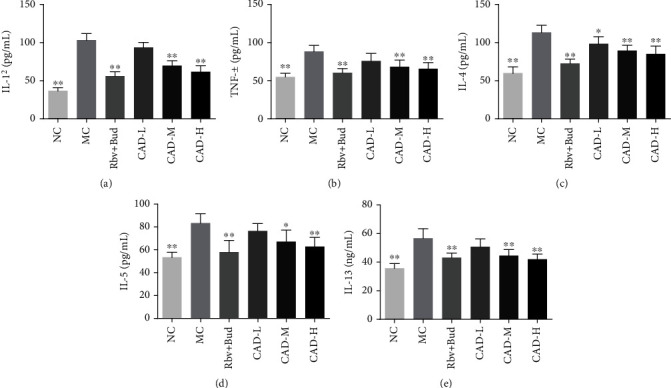
Effect of the conciliatory anti-allergic decoction on the cytokine levels in BALF of asthmatic mice induced by RSV. (a-e) Inflammatory cytokines TNF-*α*, IL-1*β*, IL-4, IL-5, and IL-13 in BALF were determined by ELISA, respectively. ^∗^*P < 0.05*, ^∗∗^*P < 0.01*, compared with the MC group.

Excessive inflammatory cell accumulation in lung tissue is considered a central clinical feature of asthma. Then, we collected BALF and counted the number of inflammatory cells. Compared with the NC group, the number of inflammatory cells in BALF of the MC group was significantly increased. In contrast, the inflammatory cells in BALF of CAD- and Rbv + Bud-treated mice were remarkably lower than in model mice (Figure 3(a)). Analysis of the numbers of neutrophils, eosinophils, and lymphocytes in BALF showed a significant increase in the MC group. The CAD-treated RSV-infected asthmatic mice, however, had significantly decreased inflammatory cells than the MC group, particularly in eosinophils (Figures 3(b)–3(d)). In addition, the results of ELISA demonstrated that the BALF levels of the five cytokines TNF-*α*, IL-1*β*, IL-4, Il-5, and IL-13 in the RSV-infected asthmatic mice were significantly higher in the MC group than in NC group. Noticeably, these cytokine levels were attenuated by CAD treatment (Figures 4(a)–4(e)).

### 3.3. CAD Ameliorated the Airway Injury and Lung Remodeling in RSV-Infected Asthmatic Mice

**Figure 5:**
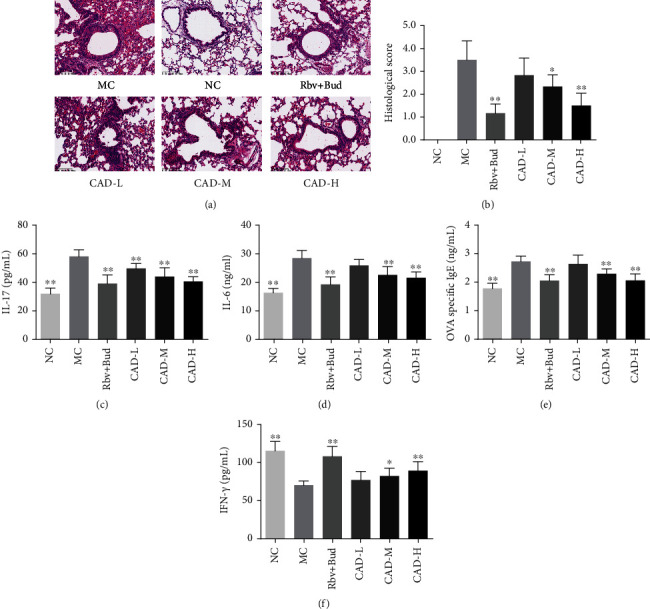
Effect of the conciliatory anti-allergic decoction on the airway inflammation in the lungs of asthmatic mice induced by RSV. (a) Representative images of HE staining sections of mouse lung tissue to assess the histopathological changes. Bar =100 *μ*m. (b) Inflammation scores of HE staining in each group. (c-f) The contents of IL-17, IL-6, OVA-specific IgE, and IFN-*γ* in serum were detected by ELISA. ^∗^*P < 0.05*, ^∗∗^*P < 0.01*, compared with the MC group.

**Figure 6:**
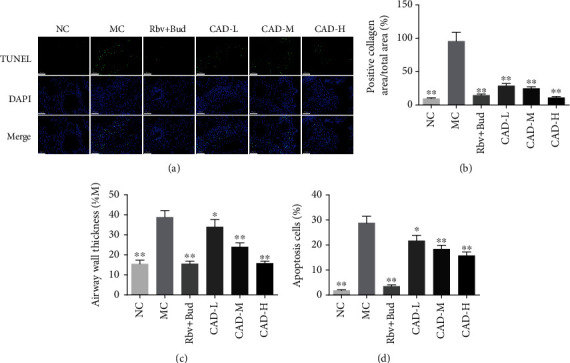
Effect of the conciliatory anti-allergic decoction on airway remodeling in asthmatic mice. (a) Representative photographs of Mason, PAS, and TUNEL-stained lung sections from asthmatic mice induced by RSV (Bar =100 *μ*m or 50 *μ*m). (b-d) The collagen deposition, airway wall thickness, and apoptotic cells were measured in asthmatic mice. ^∗^*P < 0.05*, ^∗∗^*P < 0.01*, compared with the MC group.

The lung index was significantly increased in the MC group. Conversely, in the mice treated with CAD or Rbv + Bud, lung index decreased significantly (Figure 2(d)). Also, HE staining showed obvious infiltration of inflammatory cells around the alveolar wall compared with that of the NC group. CAD or Rbv + Bud treatment remarkably attenuated inflammatory cells around the airways, which was confirmed by the inflammation score (Figures 5(a)–5(b)). Furthermore, the levels of the IL-17, IL-6, OVA-specific IgE, and IFN-*γ* in the serum after treatment of CAD were measured using an ELISA assay. As shown in Figures 5(c)–5(f), the levels of IL-17, IL-6, and OVA-specific IgE in the NC group were significantly decreased, and compared to that, increased concentrations of IL-17, IL-6, and OVA-specific IgE from MC group were found. Treatment with CAD or Rbv + Bud contributed to significantly decreasing the levels of IL-17, IL-6, and OVA-specific IgE. Besides, compared to the MC group, treatment with CAD observably increase the IFN-*γ* levels. In addition, Masson staining showed that the MC group presented an increase in collagen fiber content in airways and alveolar septa compared to the NC group, and this was noticeably reduced by CAD or Rbv + Bud treatment (Figures 6(a)–6(b)). PAS staining demonstrated that the airway wall thickness was significantly decreased in the CAD or Rbv + Bud groups compared with the MC group (Figures 6(a)–6(c)). Besides, in the normal control mice, a small number of TUNEL-positive (TUNEL^+^) cells were observed. However, a significant increase of TUNEL^+^ cells was found in the MC group, which was decreased significantly by the administration of CAD or Rbv + Bud (Figure 6(d)). Generally, these findings demonstrated that CAD treatment contributed to against airway injury and lung remodeling in RSV-infected asthmatic mice.

### 3.4. CAD Decreased Cell Apoptosis and Pyroptosis in RSV-Infected Asthmatic Mice

**Figure 7:**
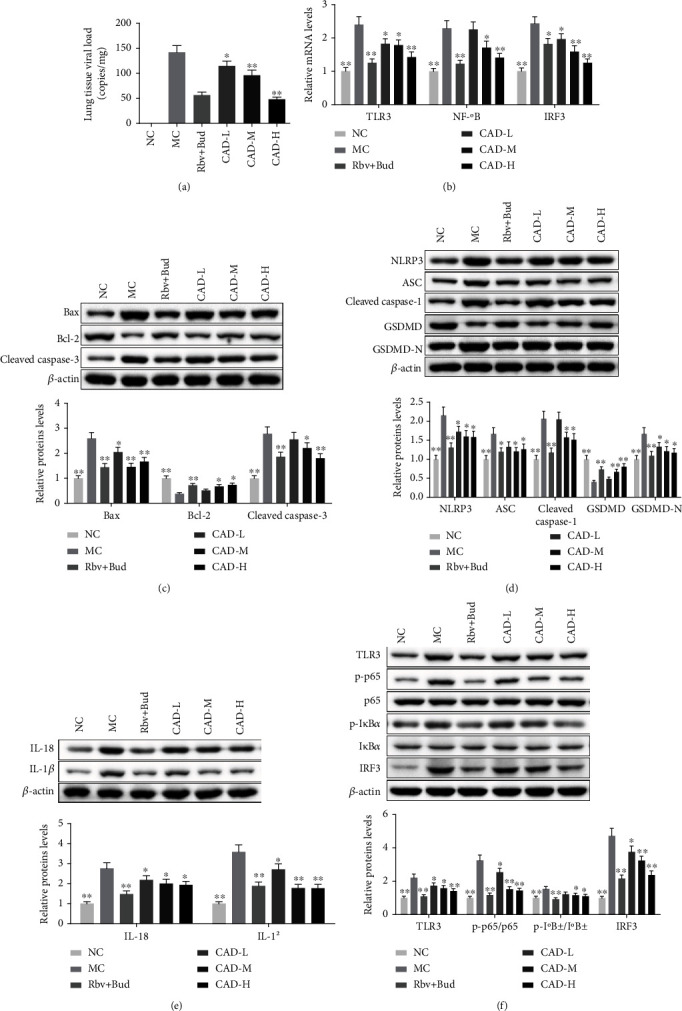
Effect of the conciliatory anti-allergic decoction on the levels of pyroptosis and TLR3/NLRP3/NF-*κ*B/IRF3 pathway-related proteins in the lungs of mice with OVA/RSV-induced asthma. (a) The lung tissue viral load was measured using a qPCR assay. (b) The mRNA levels of TLR3, NF-*κ*B, and IRF3 in lung tissues. (c) Western blot detected the expression of Bax, Bcl-2, and Cleaved caspase-3 in lung tissues. (d, e) The pyroptosis-related protein expressions were analyzed by western blotting. (f) The TLR3/NLRP3/NF-*κ*B/IRF3 pathway-related protein levels were also determined by western blotting. ^∗^*P < 0.05*, ^∗∗^*P < 0.01*, compared with the MC group.

To clarify the effect of CAD on cell apoptosis and pyroptosis in RSV-infected asthmatic mice, the qPCR and western blot assays were performed. Firstly, qPCR assay showed that asthma mice exhibited a substantial accumulation in lung tissue viral load, as well as a massive elevation in mRNA expression levels of TLR3, NF-*κ*B, and IRF3, whereas CAD down-regulated lung tissue viral load, TLR3, NF-*κ*B, and IRF3 levels in RSV-infected asthmatic mice (Figures 7(a)–7(b)). Further western blot analysis has shown that CAD decreased cell apoptosis-associated Bax and Cleaved caspase-3 protein levels, while as increased Bcl-2 protein levels in lung tissues of asthma mice (Figure 7(c)). Also, the pyroptosis-related proteins were demonstrated to be down-regulated by CAD in RSV-infected asthmatic mice using western blotting; And, whereas CAD-treated mice exhibited higher protein levels of GSDMD in lung tissues than model mice (Figures 7(d)–7(e)). Additionally, western blot analysis demonstrated that RSV-infected asthmatic mice had an increase in TLR3, p-P65, p-I*κ*B*α*, and IRF3. Conversely, CAD treatment reversed these protein levels in asthmatic mice (Figure 7(f)). These results suggested that CAD may decrease cell apoptosis and pyroptosis in lung tissues of asthma mice via inhibiting the TLR3/NLRP3/NF-*κ*B/IRF3 signaling pathway.

### 3.5. Identification of Chemical Compounds in High-Dose CAD

**Figure 8:**
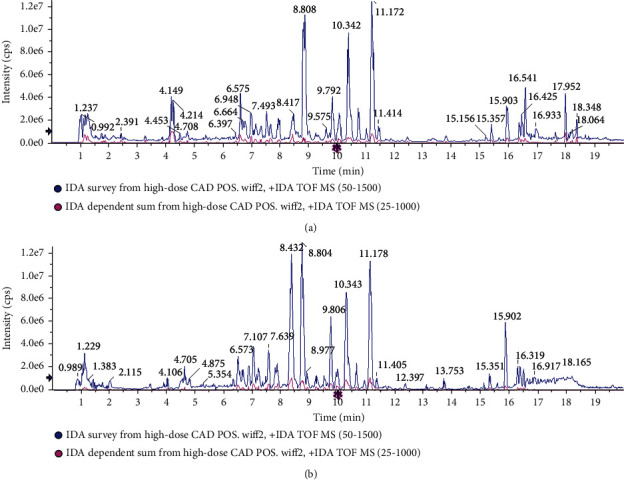
BPI chromatogram of chemical components of high-dose CAD in UPLC-Q/TOF-MS analysis. (a) BPI chromatogram of positive scan mode. (b) BPI chromatogram of negative scan mode.

The base peak ion (BPI) chromatogram of high-dose CAD in positive and negative ion modes is shown in Figure 8. As shown in Table S1, 110 chemical compositions were identified in high-dose CAD. Among these components, there were 88 unique components in high-dose CAD after deleting the duplicate items, including flavonoids, amino acids, nucleotides, alkaloids, saponins, and phenylpropanoid constituents.

### 3.6. CAD Ameliorated LPS-Induced Cell Death of 16HBE Cells

**Figure 9:**
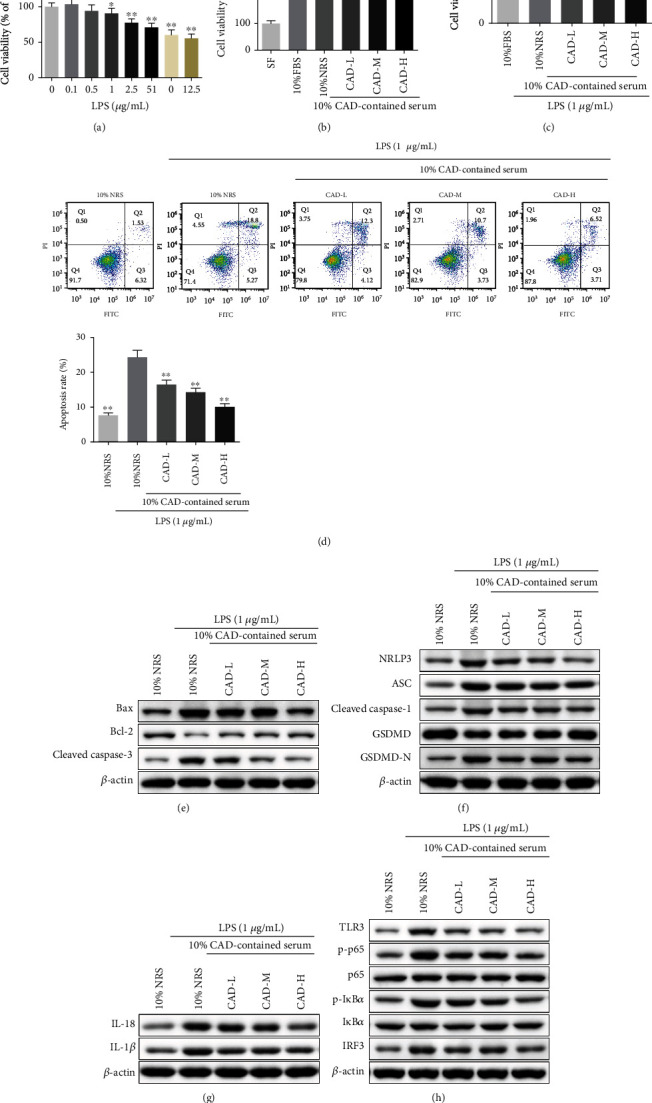
Effect of conciliatory anti-allergic decoction-contained serum on the cell survival of LPS-induced 16HBE cells. (a, b) The cell viability of 16HBE cells treated with LPS or CAD-contained serum was measured by using CCK-8 assays. (c) Cell viability of LPS-induced 16HBE cells treated with CAD-contained serum was evaluated by CCK-8 assay. (d) Representative flow cytometry results showing that the effects of CAD-contained serum on cell apoptosis in the LPS-induced 16HBE cells. (e, f, g, h) Western blotting was used to detect the expression of the apoptosis-, pyroptosis-, and TLR3/NLRP3/NF-*κ*B/IRF3 pathway-associated proteins, Bax, Bcl-2, Cleaved caspase-3, NLRP3, ASC, Cleaved caspase-1, GSDMD, GSDMD-N, IL-18, IL-1*β*, TLR3, p-P65, P65, p-I*κ*B*α*, I*κ*B*α*, and IRF3 in LPS-induced 16HBE cells after treatment of CAD-contained serum. ^∗^*P < 0.05*, ^∗∗^*P < 0.01.*

The 16HBE cells were employed to evaluate the protective effects of CAD-contained serum in vitro. As shown in Figure 8(a), the CCK-8 assay suggested that treatment with LPS at 1, 2.5, 5, 10, and 12.5 *μ*g/mL for 24 h significantly attenuated the cell viability of 16HBE cells (Figure 9(a)). Thus, we used the 1 *μ*g/mL LPS in the subsequent experiments. As shown in Figure 9(b), we found that treatment with 10% FBS, 10% NRS, and 10% CAD-contained serum promoted cell viability significantly compared with the 16HBE cells treated with SF, and no significant differences in cell viability were found between 10% FBS and 10% NRS group. For this reason, 10% NRS was selected to replace 10% FBS in the subsequent experiments. Intriguingly, 10% CAD-L, CAD-M, and CAD-H group contained serum significantly increased LPS-induced cell death of 16HBE cells (Figure 9(c)). To further investigate the role of CAD-contained serum on cell apoptosis and pyroptosis in LPS-induced 16HBE cells, cell death was estimated by using flow cytometric and western Blot assays, respectively. The results found that the apoptosis rate of 16HBE cells was increased under LPS treatment, while it was inhibited by 10% CAD-contained serum (Figure 9(d)), accompanied by a suppression of the protein expressions of Cleaved caspase-3 and Bax, increased the expression of Bcl-2 (Figure 9(e)). For cell pyroptosis, we found that the 10% CAD-contained serum could significantly inhibit ASC, NLRP3, Cleaved caspase-1, IL-18, GSDMD-N, and IL-1*β* protein levels while expression of GSDMD was promoted in LPS-induced 16HBE cells (Figures 9(f)–9(g)). To increase understanding of the mechanisms of CAD, the TLR3/NF-*κ*B/IRF3 signaling was also detected. Results revealed that 10% CAD-contained serum observably decreased the levels of TLR3, p-P65, p-I*κ*B*α*, and IRF3 in vitro (Figure 9(h)). To sum up, our data indicated that CAD-contained serum can rescue LPS-induced cell death of 16HBE cells through suppression of the TLR3/NLRP3/NF-*κ*B/IRF3 signaling pathway.

## 4. Discussions

In the current research, we investigated the effects of CAD in asthmatic mice and LPS-mediated 16HBE cells. Then, we found that CAD improved general conditions in asthma model mice, as evidenced by decreasing the number of sneezing and catching nose, respiratory rate, as well as increased tidal volume. Airway hyperresponsiveness, as one of the main characteristics of chronic asthma, is caused by airway inflammation [26, 27]. Hence, we explored the changes in airway activity immediately after the RSV-infected asthma mice model was treated with CAD, and the results have shown that CAD effectively attenuated the airway hyperresponsiveness under stimulation with different concentrations of acetylcholine. Moreover, as a primary cause of pulmonary dysfunction and airway hyperresponsiveness, airway remodeling gives rise to changes in airway structure, including airway wall thickening, collagen deposition, etc [1, 28].

Additionally, the pathological analysis demonstrated that there is a massive inflammatory cell infiltration in the lung tissue, and the thickening of the airway wall and collagen deposition in RSV-infected asthma mice were relieved by CAD. Furthermore, the release of IL-4, IL-5, and IL-13 play a central role in the initiation and development of chronic airway inflammation of asthma [29, 30]. Specifically, some evidence suggested that these cytokines are involved in eosinophil recruitment, epithelial cell apoptosis, mucus production, extracellular matrix deposition, and goblet cell hyperplasia [11, 31, 32]. Consistent with our previous findings, our results have demonstrated that the inflammatory cells, particularly neutrophils, eosinophils, and lymphocytes, were apparently increased in the asthmatic mice. Significantly, CAD relieved pulmonary inflammation and the inflammatory cell recruitment in BALF. Furthermore, it was found that the levels of TNF-*α*, IL-1*β*, IL-4, IL-5, and IL-13 in the BALF, and the levels of IL-17, IL-6, and OVA-specific IgE were observably increased in serum of RSV-infected asthma mice, while the level of IFN-*γ* was decreased. Interestingly, the results showed that CAD partially restored the imbalance between these Th1-and Th2-associated cytokines. In addition, the chemical compounds of high-dose CAD were assessed using UPLC-Q-TOF/MS analysis. Results showed that 88 unique components were found in high-dose CAD, and mainly classified into flavonoids, amino acids, nucleotides, alkaloids, saponins, and phenylpropanoid constituents. A growing body of literature suggests that the flavonoids, alkaloids, and saponins exert anti-inflammatory activities on multiple diseases [33–35], suggesting that identified components might contribute to the inhibitory activity of CAD in RSV-induced inflammation. Overall, these results revealed that CAD effectively inhibited OVA-induced airway inflammation and airway remodeling in asthmatic mice with RSV infection.

Apoptosis, or programmed cell death, is a metabolically active process characterized by nuclear fragmentation, cell membrane shrinkage, plasma membrane blebbing, and nuclear chromatin condensation [6]. Epithelial cell apoptosis is already widely accepted as a potential mechanism of asthma [36, 37]. To further investigate the effect of CAD on cell apoptosis in lung tissue, we performed a TUNEL-stained examination of the lung tissues of asthma mice. We found that CAD treatment inhibited airway cell apoptosis of lung tissues compared to the asthma mice, which was demonstrated by using TUNEL. As we have known, the Bcl-2/Bax/Cleaved caspase-3 signaling pathway has been reported in regulating cell apoptosis and survival in many diseases, including asthma [38]. Bcl-2 as an apoptosis-inhibiting protein and Bax as an apoptosis-promoting protein plays a central role in inhibiting or promoting intrinsic programmed cell death. Also, Caspase-3 is a vital protease closely related to executing the final phase of apoptosis. In a previous study, Zuo et al. found that dexamethasone-induced apoptosis of bronchial epithelial cells was significantly alleviated under Panax notoginseng saponins R1 treatment [39]. Consistent with previously published research, in this study, we have found that CAD or CAD-contained serum observably weakened the levels of Bax and Cleaved caspase-3 protein and increased Bcl-2 expressions in asthma mice and LPS-induced 16HBE cells, respectively. These results suggested that CAD inhibited the apoptosis of lung tissues in asthmatic mice and LPS-induced 16HBE cells, probably by regulating the expression of the Bcl-2/Bax/Cleaved caspase-3 signaling pathway.

Programmed cell death has been regarded as the major form of intracellular process for cell death [6]. However, because the activation of inflammation exists in the RSV-infected asthmatic mice, it could be possible that there are still other types of cell death in asthma except for apoptosis. Pyroptosis is a kind of cell lysis and pro-inflammatory cytokine release-dependent programmed cell death, regulated by NLRP3 inflammasome [11, 40]. NLRP3 inflammasome activation results in Caspase 1-dependent release of the IL-1*β* and IL-18, and the gasdermin D-mediated pyroptotic cell death [41]. Studies have suggested that pyroptosis is closely participated in the pathogenesis of many diseases, including asthma [10, 42], acute kidney injury [43], and depression [44]. Besides, we also investigated the changes of pyroptosis in OVA-induced asthma mice with RSV infection and LPS-induced 16HBE cells. Our results indicated that the RSV or LPS could induce pyroptosis in asthmatic mice and lung cells, respectively, and CAD or CAD-contained serum treatment showed therapeutic action against pyroptosis in asthma, providing a novel insight into the cell death pathways in RSV-infected asthmatic mice. Moreover, some researchers have demonstrated the crucial role of the TLR3/NF-*κ*B/IRF3 signaling pathway, which is activated by the inflammatory cytokines during the progress of virus-induced asthma [45, 46]. Ramu et al. showed that rhinovirus triggered the production of interferons and IL-33 in bronchial smooth muscle cells and asthmatic individuals were weakened by knockdown of TLR3 [47]. Also, Sugiura et al. illustrated that activation of TLR3 can affect airway remodeling by augmenting myofibroblast differentiation in asthma [48]. In our study, the levels of TLR3, p-P65, p-I*κ*B*α*, and IRF3 were clearly increased in lung tissues of asthma mice and LPS-mediated 16HBE cells. Additionally, we found that administration of CAD or CAD-contained serum inhibited the protein expressions of TLR3, p-P65, p-I*κ*B*α*, and IRF3 in tissues and 16HBE cells. These results further suggested that pyroptosis holds an important position during OVA-induced asthma with RSV-infected, and we speculate that CAD may play a therapeutic role in airway inflammation and pyroptosis by blocking TLR3/NLRP3/NF-*κ*B/IRF3 signaling.

## 5. Conclusion

Generally, these results indicated that apoptosis and pyroptosis played an important role in RSV-infected asthma mice and LPS-mediated 16HBE cells, and CAD potential inhibits the TLR3/NLRP3/NF-*κ*B/IRF3 signaling pathway and subsequently suppresses airway inflammation and pyroptosis. Therefore, the ability of CAD to improve chronic asthma makes it an effective kind of drug for asthma therapy. However, there are several other limitations in the current study. The active constituents of CAD or CAD-contained serum were not explicitly studied. Besides, more detailed basic and clinical investigations through systemic inhibition studies such as knock-down or knock-out models are required to assess whether overexpression of TLR3/NLRP3/NF-*κ*B/IRF3 signaling could reverse the protective effect of CAD on asthma.

## Data Availability

The datasets used or analyzed during the current study are available from the corresponding author on reasonable request.

## Supplementary Materials

Supplementary Materials Table S1 Chemical components information of high-dose CAD in positive and negative ion modes.
