# Supplementary material for: Conciliatory Anti-Allergic Decoction Attenuates Pyroptosis in RSV-Infected Asthmatic Mice and Lipopolysaccharide (LPS)-Induced 16HBE Cells by Inhibiting TLR3/NLRP3/NF-κB/IRF3 Signaling Pathway
Source: J Immunol Res. 2022 Sep 29;2022:1800401. doi: 10.1155/2022/1800401 (PMC9537000; doi:10.1155/2022/1800401)
Supplement: Supplementary Materials — Table S1 Chemical components information of high-dose CAD in positive and negative ion modes. [file 1800401.f1.docx]

**Table S1 Chemical components information of high-dose CAD in positive and negative ion modes**

| No. | Component Name | Adduct | Area | Retention Time | Formula | Precursor Mass | Found At Mass | Mass Error (ppm) | Library Score |
| --- | --- | --- | --- | --- | --- | --- | --- | --- | --- |
| 1 | L(+)-Arginine | [M+H] | 5890000 | 1.12 | C6H14N4O2 | 175.119 | 175.119 | 0.1 | 94.5 |
| 2 | Histidine | [M+H] | 22890 | 1.14 | C6H9N3O2 | 156.077 | 156.0771 | 2.2 | 98.8 |
| 3 | Glutamic acid | [M+H] | 193800 | 1.17 | C5H9NO4 | 148.06 | 148.0606 | 0.9 | 97.1 |
| 4 | Betaine | [M+H] | 613500 | 1.18 | C5H11NO2 | 118.086 | 118.0863 | 0.1 | 100 |
| 5 | Trigonelline | [M+H] | 557800 | 1.21 | C7H7NO2 | 138.055 | 138.0547 | -1.6 | 94.9 |
| 6 | Proline | [M+H] | 3126000 | 1.23 | C5H9NO2 | 116.071 | 116.0705 | -0.5 | 97.8 |
| 7 | Nicotinic acid | [M+H] | 70990 | 1.76 | C6H5NO2 | 124.039 | 124.0394 | 0.7 | 99.4 |
| 8 | Nicotinamide | [M+H] | 101300 | 1.83 | C6H6N2O | 123.055 | 123.0553 | 0.5 | 99.5 |
| 9 | 6-Hydroxypurine | [M+H] | 228600 | 1.94 | C5H4N4O | 137.046 | 137.0457 | -1 | 99.3 |
| 10 | Adenine | [M+H] | 384300 | 2.4 | C5H5N5 | 136.062 | 136.0617 | -0.3 | 94.8 |
| 11 | Adenosine | [M+H] | 1742000 | 2.4 | C10H13N5O4 | 268.104 | 268.1038 | -0.9 | 98 |
| 12 | Cordycepin | [M+H] | 33500 | 2.46 | C10H13N5O3 | 252.109 | 252.109 | -0.4 | 100 |
| 13 | Guanosine | [M+H] | 234300 | 2.51 | C10H13N5O5 | 284.099 | 284.0989 | -0.2 | 100 |
| 14 | Phenylalanine | [M+H] | 494700 | 3.24 | C9H11NO2 | 166.086 | 166.0861 | -0.9 | 99.8 |
| 15 | 5-Hydroxymethylfurfural | [M+H] | 148300 | 3.36 | C6H6O3 | 127.039 | 127.0389 | -0.7 | 98.7 |
| 16 | Griffonilide | [M+H] | 15970 | 3.45 | C8H8O4 | 169.05 | 169.0495 | -0.2 | 76.7 |
| 17 | Esculetin | [M+H] | 37530 | 3.51 | C9H6O4 | 179.034 | 179.0337 | -0.8 | 93.9 |
| 18 | Pseudoephedrine | [M+H] | 3427000 | 4.17 | C10H15NO | 166.123 | 166.1225 | -1 | 99.3 |
| 19 | Syringin | [M+H] | 20670 | 4.67 | C17H24O9 | 390.176 | 390.1756 | -0.8 | 96.8 |
| 20 | Protocatechuic Aldehyde | [M+H] | 75790 | 4.69 | C7H6O3 | 139.039 | 139.0389 | -0.7 | 97.8 |
| 21 | Chlorogenic acid | [M+H] | 2242000 | 4.72 | C16H18O9 | 355.102 | 355.102 | -1 | 99.6 |
| 22 | Epicatechin | [M+H] | 34580 | 4.78 | C15H14O6 | 291.086 | 291.0862 | -0.5 | 87.5 |
| 23 | Fraxin | [M+H] | 12290 | 5.26 | C16H18O10 | 371.097 | 371.0976 | 1 | 81.1 |
| 24 | 8-Epiloganic acid | [M+H] | 25290 | 5.41 | C16H24O10 | 377.144 | 377.1449 | 1.9 | 90.8 |
| 25 | Schaftoside | [M+H] | 1107000 | 5.79 | C26H28O14 | 565.155 | 565.1544 | -1.4 | 93.5 |
| 26 | Eleutheroside E | [M+H] | 17960 | 5.98 | C34H46O18 | 760.302 | 760.3019 | -0.4 | 94.9 |
| 27 | Vitexin-2-O-rhamnoside | [M+H] | 773600 | 6.34 | C27H30O14 | 579.171 | 579.1703 | -0.9 | 97.6 |
| 28 | Rutin | [M+H] | 1314000 | 6.4 | C27H30O16 | 611.161 | 611.1601 | -0.9 | 97.2 |
| 29 | Ellagic Acid | [M+H] | 1310 | 6.69 | C14H6O8 | 303.014 | 303.0135 | 0 | 91.9 |
| 30 | Isoquercitrin | [M+H] | 246500 | 6.7 | C21H20O12 | 465.103 | 465.1024 | -0.8 | 99.6 |
| 31 | Hyperin | [M+H] | 246500 | 6.7 | C21H20O12 | 465.103 | 465.1024 | -0.8 | 100 |
| 32 | Liquiritigenin | [M+H] | 1512000 | 6.74 | C15H12O4 | 257.081 | 257.0805 | -1.2 | 93.1 |
| 33 | Scutellarin | [M+H] | 1192000 | 6.75 | C21H18O12 | 463.087 | 463.0867 | -1 | 99.5 |
| 34 | Isoscopoletin | [M+H] | 18260 | 6.8 | C10H8O4 | 193.05 | 193.0496 | 0.2 | 98.1 |
| 35 | Isoferulic acid | [M+H] | 7313 | 6.85 | C10H10O4 | 195.065 | 195.0654 | 1 | 99.2 |
| 36 | Peimisine | [M+H] | 520000 | 7.19 | C27H41NO3 | 428.316 | 428.3153 | -1.5 | 89.5 |
| 37 | Peimine | [M+H] | 7064000 | 7.49 | C27H45NO3 | 432.347 | 432.3467 | -1.1 | 100 |
| 38 | Puerarin | [M+H] | 383300 | 7.56 | C21H20O9 | 417.118 | 417.1176 | -0.9 | 95.4 |
| 39 | Isochlorogenic acid A | [M+H] | 1662000 | 7.63 | C25H24O12 | 517.134 | 517.1334 | -1.3 | 99.2 |
| 40 | Peiminine | [M+H] | 4357000 | 7.89 | C27H43NO3 | 430.332 | 430.3309 | -1.7 | 100 |
| 41 | Liquiritin | [M+H] | 517800 | 8.57 | C21H22O9 | 419.134 | 419.1332 | -1.1 | 99.7 |
| 42 | Baicalin | [M+H] | 58210000 | 8.81 | C21H18O11 | 447.092 | 447.0915 | -1.6 | 97 |
| 43 | Apigenin-7-glucoside | [M+H] | 107700 | 8.86 | C21H20O10 | 433.113 | 433.1133 | 0.9 | 99.3 |
| 44 | Ononin | [M+H] | 745800 | 8.91 | C22H22O9 | 431.134 | 431.1332 | -1 | 98.6 |
| 45 | Wogonin 7-O-glucuronide | [M+H] | 50730000 | 11.17 | C22H20O11 | 461.108 | 461.107 | -1.8 | 100 |
| 46 | Isorhamnetin | [M+H] | 20200 | 12.83 | C16H12O7 | 317.066 | 317.0653 | -0.9 | 70.4 |
| 47 | Cardamonin | [M+H] | 25440 | 13.16 | C16H14O4 | 271.096 | 271.0963 | -0.7 | 96.6 |
| 48 | Baicalein | [M+H] | 1467000 | 13.74 | C15H10O5 | 271.06 | 271.0598 | -1.1 | 81 |
| 49 | Glycyrrhizic acid | [M+H] | 5202000 | 15.91 | C42H62O16 | 823.411 | 823.4103 | -1 | 97.2 |
| 50 | Wogonin | [M+H] | 3734000 | 16.34 | C16H12O5 | 285.076 | 285.0754 | -1.1 | 93.7 |
| 51 | Saikosaponin B2 | [M+H] | 229800 | 16.42 | C42H68O13 | 781.473 | 781.4716 | -2.1 | 99.7 |
| 52 | Saikosaponin D | [M+H] | 229800 | 16.42 | C42H68O13 | 781.473 | 781.4716 | -2.1 | 99.7 |
| 53 | Wilforlide A | [M+H] | 1437000 | 16.42 | C30H46O3 | 455.352 | 455.3506 | -3.1 | 73 |
| 54 | Chrysin | [M+H] | 122400 | 16.44 | C15H10O4 | 255.065 | 255.0648 | -1.4 | 94.8 |
| 55 | Dihydrotanshinone I | [M+H] | 436800 | 17.55 | C18H14O3 | 279.102 | 279.1008 | -2.9 | 92.9 |
| 56 | Tanshinone I | [M+H] | 91880 | 17.79 | C18H12O3 | 277.086 | 277.0852 | -2.5 | 89.9 |
| 57 | Cryptotanshinone | [M+H] | 7770000 | 17.95 | C19H20O3 | 297.149 | 297.1471 | -4.8 | 93.8 |
| 58 | Tanshinone IIA | [M+H] | 3333000 | 18.35 | C19H18O3 | 295.133 | 295.1318 | -3.6 | 73.2 |
| 59 | Histidine | [M-H] | 25910 | 1.09 | C6H9N3O2 | 154.062 | 154.0622 | -0.2 | 92.2 |
| 60 | Arginine | [M-H] | 212000 | 1.1 | C6H14N4O2 | 173.104 | 173.1041 | -1.5 | 99.5 |
| 61 | Sorbitol | [M-H] | 50120 | 1.14 | C6H14O6 | 181.072 | 181.072 | 1.4 | 92.2 |
| 62 | Glutamic acid | [M-H] | 62640 | 1.14 | C5H9NO4 | 146.046 | 146.0461 | 1.8 | 98.5 |
| 63 | D-Galactose | [M-H] | 208100 | 1.19 | C6H12O6 | 179.056 | 179.0563 | 0.8 | 95.7 |
| 64 | D-(+)-Glucose | [M-H] | 208100 | 1.19 | C6H12O6 | 179.056 | 179.0563 | 0.8 | 95.7 |
| 65 | D-(+)-Mannose | [M-H] | 208100 | 1.19 | C6H12O6 | 179.056 | 179.0563 | 0.8 | 94 |
| 66 | Citric acid | [M-H] | 1278000 | 2.01 | C6H8O7 | 191.02 | 191.0196 | -0.9 | 99 |
| 67 | Succinic acid | [M-H] | 96040 | 2.33 | C4H6O4 | 117.019 | 117.0194 | 0.9 | 94.8 |
| 68 | Adenine | [M-H] | 59950 | 2.39 | C5H5N5 | 134.047 | 134.0474 | 1 | 78.4 |
| 69 | Adenosine | [M-H] | 16840 | 2.39 | C10H13N5O4 | 266.089 | 266.0896 | 0.5 | 91.1 |
| 70 | Guanosine | [M-H] | 369600 | 2.51 | C10H13N5O5 | 282.084 | 282.0841 | -1 | 99.2 |
| 71 | Gallic acid | [M-H] | 2923 | 2.7 | C7H6O5 | 169.014 | 169.0146 | 1.9 | 85.1 |
| 72 | Phenylalanine | [M-H] | 143100 | 3.23 | C9H11NO2 | 164.072 | 164.0716 | -0.8 | 94.3 |
| 73 | Danshensu | [M-H] | 891600 | 3.51 | C9H10O5 | 197.046 | 197.0455 | -0.4 | 98.8 |
| 74 | L-Tryptophan | [M-H] | 329400 | 4.22 | C11H12N2O2 | 203.083 | 203.0824 | -0.9 | 96.9 |
| 75 | Protocatechuic Aldehyde | [M-H] | 774000 | 4.69 | C7H6O3 | 137.024 | 137.0244 | -0.4 | 97.8 |
| 76 | Chlorogenic acid | [M-H] | 2931000 | 4.71 | C16H18O9 | 353.088 | 353.0875 | -1 | 99.4 |
| 77 | Quinic acid | [M-H] | 1176000 | 4.72 | C7H12O6 | 191.056 | 191.0562 | 0.3 | 73.5 |
| 78 | Catechin | [M-H] | 103000 | 4.79 | C15H14O6 | 289.072 | 289.0718 | 0.1 | 90.4 |
| 79 | Caffeic acid | [M-H] | 460200 | 5.29 | C9H8O4 | 179.035 | 179.035 | 0 | 72.1 |
| 80 | Sibiricose A5 | [M-H] | 138500 | 5.45 | C22H30O14 | 517.156 | 517.1566 | 0.6 | 94.1 |
| 81 | Eleutheroside E | [M-H] | 83320 | 5.98 | C34H46O18 | 787.267 | 787.2666 | 0 | 100 |
| 82 | Vitexin-2-O-rhamnoside | [M-H] | 854500 | 6.34 | C27H30O14 | 577.156 | 577.1565 | 0.4 | 97.9 |
| 83 | p-Coumaric acid | [M-H] | 12150 | 6.38 | C9H8O3 | 163.04 | 163.0403 | 1.2 | 98.3 |
| 84 | Rutin | [M-H] | 2400000 | 6.41 | C27H30O16 | 609.146 | 609.1457 | -0.7 | 99.2 |
| 85 | Hyperin | [M-H] | 945400 | 6.7 | C21H20O12 | 463.088 | 463.0882 | -0.1 | 97.6 |
| 86 | Liquiritin | [M-H] | 3979000 | 6.74 | C21H22O9 | 417.119 | 417.119 | -0.2 | 96.9 |
| 87 | Aloin, Barbaloin | [M-H] | 3979000 | 6.74 | C21H22O9 | 417.119 | 417.119 | -0.2 | 96.9 |
| 88 | Acteoside | [M-H] | 58490 | 6.75 | C29H36O15 | 623.198 | 623.1976 | -0.9 | 98.5 |
| 89 | Scutellarin | [M-H] | 375100 | 6.76 | C21H18O12 | 461.073 | 461.0724 | -0.3 | 100 |
| 90 | Isochlorogenic acid A/B/C | [M-H] | 10720000 | 7.11 | C25H24O12 | 515.119 | 515.1192 | -0.7 | 92.3 |
| 91 | Puerarin | [M-H] | 282700 | 7.55 | C21H20O9 | 415.103 | 415.1036 | 0.4 | 95.9 |
| 92 | Rosmarinic acid | [M-H] | 4334000 | 7.87 | C18H16O8 | 359.077 | 359.0771 | -0.4 | 99.6 |
| 93 | Salvianolic acid A | [M-H] | 4326000 | 7.95 | C26H22O10 | 493.114 | 493.1139 | -0.3 | 96.5 |
| 94 | Scutellarein | [M-H] | 473000 | 8.16 | C15H10O6 | 285.04 | 285.0404 | -0.3 | 92.8 |
| 95 | Salvianolic acid B | [M-H] | 40370000 | 8.43 | C36H30O16 | 717.146 | 717.1457 | -0.5 | 92.9 |
| 96 | Ononin +HCOOH | [M-H] | 171800 | 8.93 | C22H22O9 | 475.125 | 475.1247 | 0.3 | 99.5 |
| 97 | Liquiritigenin | [M-H] | 135900 | 9.5 | C15H12O4 | 255.066 | 255.0664 | 0.4 | 90.1 |
| 98 | Cistanoside D | [M-H] | 72890 | 9.68 | C31H40O15 | 651.229 | 651.2298 | 0.6 | 97.6 |
| 99 | Baicalin | [M-H] | 8690000 | 9.81 | C21H18O11 | 445.078 | 445.0778 | 0.4 | 96.9 |
| 100 | Wogonin 7-O-glucuronide | [M-H] | 15280000 | 11.18 | C22H20O11 | 459.093 | 459.0931 | -0.4 | 99 |
| 101 | Isorhamnetin | [M-H] | 54280 | 12.83 | C16H12O7 | 315.051 | 315.0514 | 1.3 | 91.6 |
| 102 | Heterophyllin B | [M-H] | 342000 | 13.29 | C40H58N8O8 | 777.43 | 777.4312 | 0.9 | 100 |
| 103 | Formononetin | [M-H] | 37060 | 14.95 | C16H12O4 | 267.066 | 267.0666 | 1.4 | 94.9 |
| 104 | Saikosaponin C | [M-H] | 534600 | 15.15 | C48H78O17 | 971.522 | 971.5224 | 0.3 | 96.8 |
| 105 | Glycyrrhizic acid | [M-H] | 12600000 | 15.9 | C42H62O16 | 821.397 | 821.3972 | 0.9 | 97 |
| 106  107 | Saikosaponin A | [M-H] | 4245000 | 16.41 | C42H68O13 | 825.464 | 825.465 | 0.9 | 98.1 |
|  | Saikosaponin D |  |  |  |  |  |  |  |  |
| 108 | Chrysin | [M-H] | 52650 | 16.44 | C15H10O4 | 253.051 | 253.0512 | 2.4 | 83.6 |
| 109 | Chrysosplenetin B | [M-H] | 4586000 | 16.53 | C19H18O8 | 373.093 | 373.0931 | 0.7 | 94.4 |
| 110 | Eupatilin | [M-H] | 248400 | 16.81 | C18H16O7 | 343.082 | 343.0827 | 1.1 | 96.7 |
